# Determinants of Mammal and Bird Species Richness in China Based on Habitat Groups

**DOI:** 10.1371/journal.pone.0143996

**Published:** 2015-12-02

**Authors:** Haigen Xu, Mingchang Cao, Jun Wu, Lei Cai, Hui Ding, Juncheng Lei, Yi Wu, Peng Cui, Lian Chen, Zhifang Le, Yun Cao

**Affiliations:** 1 Nanjing Institute of Environmental Sciences, Ministry of Environmental Protection, Nanjing 210042, China; 2 Department of Natural Ecology Conservation, Ministry of Environmental Protection, Beijing 100035, China; 3 College of Forest Resources and Environment, Nanjing Forestry University, Nanjing 210037, China; 4 Department of Biology, Nanjing University, Nanjing 210093, China; University of Waikato (National Institute of Water and Atmospheric Research), NEW ZEALAND

## Abstract

Understanding the spatial patterns in species richness is a central issue in macroecology and biogeography. Analyses that have traditionally focused on overall species richness limit the generality and depth of inference. Spatial patterns of species richness and the mechanisms that underpin them in China remain poorly documented. We created a database of the distribution of 580 mammal species and 849 resident bird species from 2376 counties in China and established spatial linear models to identify the determinants of species richness and test the roles of five hypotheses for overall mammals and resident birds and the 11 habitat groups among the two taxa. Our result showed that elevation variability was the most important determinant of species richness of overall mammal and bird species. It is indicated that the most prominent predictors of species richness varied among different habitat groups: elevation variability for forest and shrub mammals and birds, temperature annual range for grassland and desert mammals and wetland birds, net primary productivity for farmland mammals, maximum temperature of the warmest month for cave mammals, and precipitation of the driest quarter for grassland and desert birds. Noteworthily, main land cover type was also found to obviously influence mammal and bird species richness in forests, shrubs and wetlands under the disturbance of intensified human activities. Our findings revealed a substantial divergence in the species richness patterns among different habitat groups and highlighted the group-specific and disparate environmental associations that underpin them. As we demonstrate, a focus on overall species richness alone might lead to incomplete or misguided understanding of spatial patterns. Conservation priorities that consider a broad spectrum of habitat groups will be more successful in safeguarding the multiple services of biodiversity.

## Introduction

Understanding spatial patterns in species richness is a central issue in macroecology and biogeography [1–5]. Spatial patterns in species richness are essentially generated by speciation, colonization and extinction processes along ecological and historical time scales [6] and correlated with variation in climate, topography and history [4, 7–15]. Since the 19^th^ century, ecologists and biogeographers have been exploring the underlying mechanisms of species richness pattern across the earth, and this issue has triggered hot scientific debates for a long time [7–8, 16–19]. In the context of global climate change, the studies of species richness pattern are likely to offer reliable evidences for the prediction of the possible response of biodiversity to the environmental change, and have greater implication for biodiversity conservation [2].

To explain the spatial patterns of species richness, more than 100 explanatory hypotheses have been proposed to date [8, 20]. Among them, such hypotheses are at the core of current studies, e.g., the energy hypothesis, the environmental stability hypothesis and the habitat heterogeneity hypothesis. The energy hypothesis can be classified into three versions: The “water-energy dynamics” version states that areas with higher water-energy dynamics harbor higher species richness [3, 21]. The “ambient energy” version was established based on the physiological requirements of organisms [3, 22] and considers heat as the predictor of species richness gradients [9]. The “productivity” version asserts that species richness increases with productivity [22]. Accordingly, animal species richness can be driven by resource availability, e.g., plant biomass for herbivores, herbivore biomass for predators [3, 22]. The environmental stability hypothesis claims that a stable environment can accelerate species specialization and ecological niche diversification, which may further cause an increase in species richness [21, 23]. The habitat heterogeneity hypothesis posits that species richness gradients are correlated with heterogeneity in habitats [4], because variability in elevation, landscape or vegetation can create diverse habitats for co-existing species and thereby lead to higher species richness [21]. However, the explanatory power of different hypotheses and their relative roles in explaining variation of species richness lack scrutiny [4, 8].

Some previous studies indicate that a unified mechanism associated with environmental determinants can not generally be proposed when explaining species richness at different scales [6, 10, 24–26]. Species richness patterns across the earth are the consequences of the multiple interaction of a multitude of biotic and environmental variables. Analyses that focus on overall species richness may represent the majority of species among multiple taxa particularly based on the geographical distribution and thus bring the obstacle to the generality and depth of inference [27]. In recent years, some ecologists initiated to make research by partitioning variation in species richness of one or several specific taxa, e.g., birds in North America [6], bats in the entire New World [28], and macroinvertebrates in Finland [29]. Though some advances have been made currently, it still lacks a systematic study of species richness pattern and the related potential mechanisms in various habitats across a broad geographical range.

**Fig 1:**
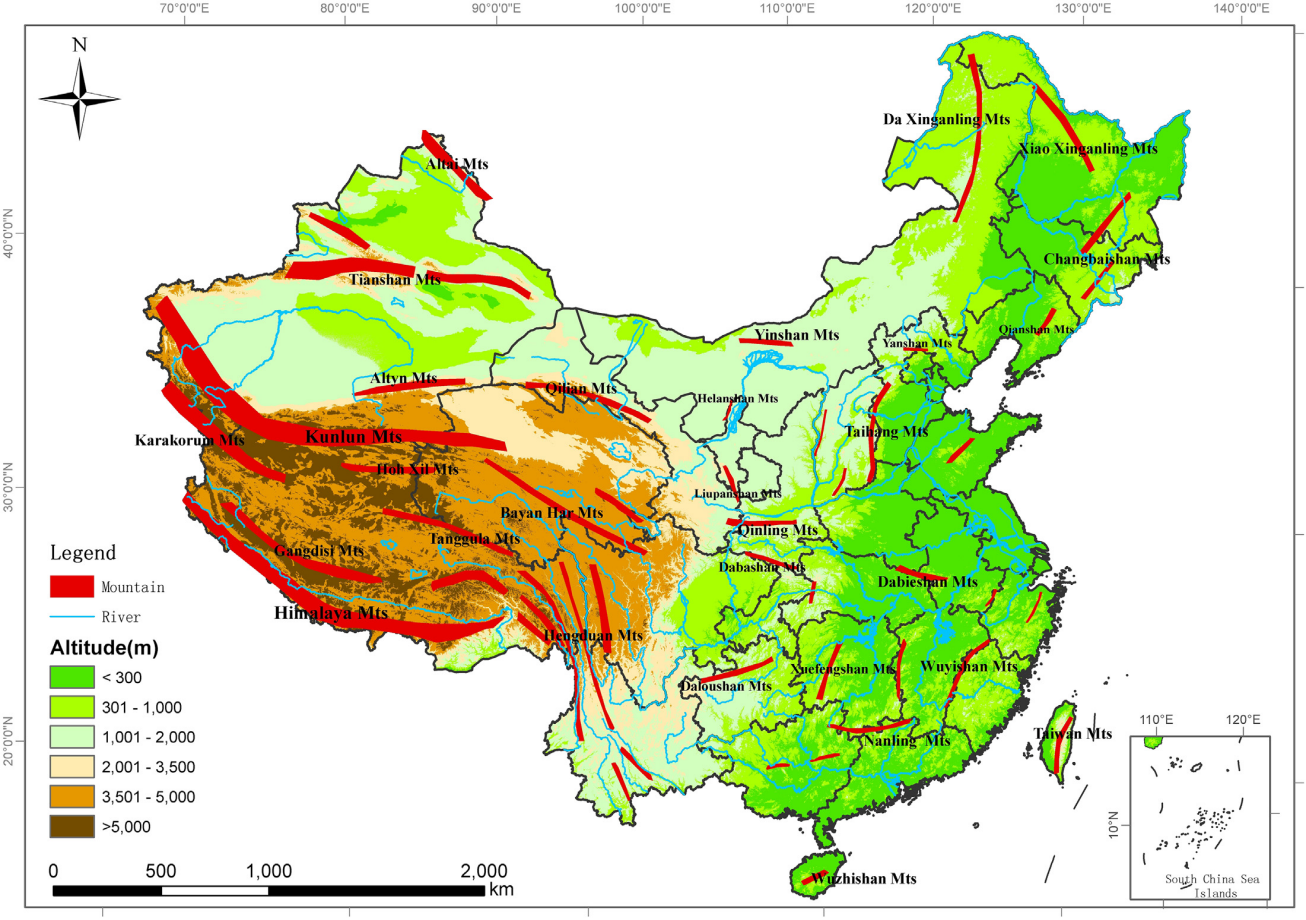
Sketch map of China showing elevation and major mountain ranges. The names of major mountain ranges were derived from Shen (2001). The inset in the right bottom of the figure shows the south boundary of China, including all islands in the South China Sea. This figure was used only for illustrative purposes.

China is one of several ‘mega-diversity’ countries in the world [30]. It covers a huge geographical area (9.6 million km^2^), from tropical to boreal zones, from a very low altitude (156 m below sea level) to the highest mountain in the world (Mount Everest) (Fig 1) [31]. In particular, most of the different types of biomes on the earth are found in China, which exhibits as a prerequisite for rich biodiversity especially the diverse mammals and birds [12] and offers an opportunity to study spatial patterns of the species richness and the mechanisms that underpin them. In recent years, some considerable progresses have been made in empirical descriptions of its biogeography [32–34] and quantitative analyses of some taxa [5, 12, 35–40]. However, there are few quantitative analyses at a finer resolution based on a complete database of all mammals and birds across China.

For decades, biodiversity surveys have been carried out in various regions of China. Based on the survey records and relevant literature, we comprehensively collected the data on the geographical distribution of species at the county level in the terrestrial and inland water ecosystems of China. In this study, we compiled a database of the geographical distribution of 580 mammal species and 849 resident bird species from 2376 counties in China. By partitioning the two taxa into 11 habitat groups, we aim to (i) identify the most dominant determinants of species richness of overall mammals and resident birds and that among different habitat groups; (ii) test the roles of five hypotheses [i.e. the water-energy hypothesis, the ambient energy (temperature) hypothesis, the productivity hypothesis, the environmental stability hypothesis and the habitat heterogeneity hypothesis] in explaining variation of species richness of overall mammals and resident birds and that among different habitat groups; and (iii) explore the implication of our analysis based on habitat groups to biodiversity conservation across China.

## Materials and Methods

### Species richness data

We compiled a database of the geographical distribution of 580 mammal species and 849 resident bird species from 2376 counties in the terrestrial and inland water ecosystems of China (see S1 and S2 Appendices), which account for nearly all the mammal and resident bird species native to China [41–42]. We excluded species in marine ecosystems, cultivated or bred species in zoos or farms, and exotic species. In addition, migratory birds were excluded in this study since this group shows the complex association with environment variables. To get a finer resolution and facilitate the future work of biodiversity conservation in China, we mainly used ‘county’ as the basic assessment unit in this study. In addition, we treated the following units respectively as an assessment unit, i.e., a municipality, a capital city in a province or autonomous region, a city at prefectural level, and a special administrative region (e.g., Hong Kong, Macau) since the distribution data were recorded at the above levels instead of county-level. To sum up, 2376 assessment units (thereafter named as counties) were used in this study. The checklist of mammal and bird species in the database was derived from the complete studies [42–43]. The information of species distribution in the database was collected from three sources: (i) more than 400 literature on the distribution of birds and mammals from 1970 to 2012 (80%), e.g. Fauna Sinicae, provincial monographs on birds and mammals [42–44]; (ii) collection information of specimens in herbaria of more than 20 institutes and universities (15%); and (iii) field survey records by experts specialized in taxonomy or biodiversity from over 30 institutes in China (5%). To reduce sampling bias and obtain more accurate distribution, more than 20 taxonomists specialized in the study of mammal and bird taxa from China were invited to review the spatial distribution of each species in the database. To elucidate the species richness in different habitats, we classified mammal species into six habitat groups and resident bird species into five habitat groups according to their habitat types (see S1 and S2 Appendices). The habitat types of mammals and resident birds were compiled based on Fauna Sinicae [44]. Generalist species were categorized into several habitat groups if they occupy more than one habitat type.

### Environmental data

Five main hypotheses that had been previously used to explain broad-scale species richness gradients [3–5, 10, 27] were tested in this study, including the energy hypotheses (water-energy dynamics, ambient energy and productivity), the environmental stability hypothesis and the habitat heterogeneity hypothesis (Table 1). We selected 19 environmental variables for above hypotheses and relevant versions as follows: (i) water: mean annual precipitation, precipitation of the wettest quarter, precipitation of the driest quarter, and mean annual dryness; (ii) ambient energy (temperature): mean annual temperature, maximum temperature of the warmest month, minimum temperature of the coldest month, and annual potential evapotranspiration; (iii) productivity: annual actual evapotranspiration, net primary productivity, and normalized difference vegetation index; (iv) environmental stability: mean diurnal range, temperature seasonality, temperature annual range, and precipitation seasonality; and (v) habitat heterogeneity: elevation variability, mean elevation, main land cover type, and number of land cover types (Table 1). Although species richness gradients are also attributed to differences in evolutionary history according to the historical hypothesis [23, 45], we did not test the historical hypothesis in this study as it was difficult to evaluate the historical effects in plurality like other variables and incorporate historical factors into regression models [46]. Data sources of environmental variables and the responding resolutions are shown in Table 1. Overall, values of environmental variables in a county were calculated as the average value of the variables among all pixels in the county. Elevation variability was calculated as the maximum minus the minimum elevation recorded in a county. Main type of land cover was estimated by the majority of land cover type in a county. Mean annual dryness was calculated as the mean annual precipitation divided by the annual potential evapotranspiration [47].

**Table 1:** Five main hypotheses explaining species richness patterns.

Hypotheses and variables	Unit	Time scale (year)	Resolution	Data source	Data transfor-mation
**Water**					
1. Mean annual precipitation	mm	1950–2000	30"×30"	WorldClim-Global Climate Data (http://www.worldclim.org)	log10
2. Precipitation of the wettest quarter	mm	1950–2000	30"×30"	The same as variable 1	log10
3. Precipitation of the driest quarter	mm	1950–2000	30"×30"	The same as variable 1	log10
4. Mean annual dryness	-	1950–2000	30"×30"	Website of the Consortium for Spatial Information (CGIAR-CSI) of the Consultative Group on International Agricultural Research (http://www.cgiar-csi.org)	log10
**Ambient energy (temperature)**					
5. Mean annual temperature	0.1°C	1950–2000	30"×30"	The same as variable 1	log10
6. Maximum temperature of the warmest month	0.1°C	1950–2000	30"×30"	The same as variable 1	log10
7. Minimum temperature of the coldest month	0.1°C	1950–2000	30"×30"	The same as variable 1	log10
8. Annual potential evapotranspiration	mm	1950–2000	30"×30"	The same as variable 4	log10
**Productivity**					
9. Annual actual evapotranspiration	mm	1961–1990	0.5°×0.5°	UNEP website (http://www.grid.unep.ch/data/download/gnv183.zip)	log10
10. Net primary productivity	gC·m^-2^·a^-1^	2000–2011	30"×30"	NASA/EOS Project of the University of Montana (http://www.ntsg.umt.edu/project/mod17#data-product)	log10
11. Normalized difference vegetation index	-	1982–2006	250m×250m	SRTM90 of Global Land Cover Facility (http://glcf.umd.edu/data/)	log10
**Environmental stability**					
12. Mean diurnal range	0.1°C	1950–2000	30"×30"	The same as variable 1	log10
13. Temperature seasonality	-	1950–2000	30"×30"	The same as variable 1	log10
14. Temperature annual range	0.1°C	1950–2000	30"×30"	The same as variable 1	log10
15. Precipitation seasonality	-	1950–2000	30"×30"	The same as variable 1	log10
**Habitat heterogeneity**					
16. Elevation variability	m	-	90m×90m	SRTM90 of Global Land Cover Facility (http://glcf.umd.edu/data/)	log10
17. Mean elevation	m	-	90m×90m	The same as variable 16	log10
18. Main land cover type	-	-	30"×30"	The European Space Agency (http://bioval.jrc.ec.europa.eu/productsglc2000/glc2000.php)	-
19. Number of land cover types	-	-	30"×30"	The same as variable 18	-

### Statistical analyses

We used multivariate models to test hypotheses explaining species richness patterns as follows (S1 Fig):

#### Data Processing

Counties in China vary in size (mean: 3908.7km^2^; standard deviation: 9287.6km^2^), which might have effects on species richness. We regressed species richness on county area (both variables were log_10_-transformed), and obtained the residuals of species richness [48]. The residuals of species richness (S3 Appendix) were used to avoid the effects of area [49]. County area and environmental variables were log10-transformed and the residuals of species richness were used in all analyses unless otherwise stated. Statistical analyses were carried out using the statistical software R 2.15 unless otherwise stated [50, 51].

#### Variable Selection

Multicollinearity of explanatory variables can cause failures in regression analyses [50]. We successively took two approaches to select the variables for model analyses to exclude multicollinearity among the variables. First, we performed Spearman (two-sided) correlation analysis (S1 Table) to avoid multicollinearity between any two variables in each category. In addition, we also calculated the deviance of variables in univariate regression models. If the correlation coefficient between them was >0.7, we removed strongly intercorrelated variables and retained the variables that explained more deviance in univariate regression models [4, 52–53]. For example, if elevation variability and mean elevation were highly correlated (r>0.7) and the explained deviance of elevation variability in univariate models was larger than that of mean elevation, we dropped mean elevation from further analysis. Thus, we selected a set of variables from each category for the next analysis (S2 Table). In the second step, we performed hierarchical partitioning analysis with the combination of selected predictors from each category to screen out the predictors that have the most independent effects on the residuals of species richness since this method can overcome multicollinearity among the variables [50]. In the hierarchical partitioning, all possible models were considered in a hierarchical multivariate regression setting to jointly identify most possible predictors. The increased goodness-of-fit was calculated in each model with a particular variable compared to the equivalent model without that variable, and the improvement in the fit was averaged across all possible models with that predictor included [54]. Then, we got a list of predictors and their independent and joint effects on the residuals of species richness [54, 55]. A 1000-randomization procedure was carried out to test the statistical significance of the independent effects of each predictor, which was expressed as a z-score [50]. A z-score greater than or equal to 1.65 is statistically significant at p<0.05. Finally, we selected the top six predictors according to z-score because they had distinctly larger independent effects than any other variables and avoided the multicollinearity among variables across the categories (S3 Table).

#### Model Selection

First, we used generalized linear models (GLM) to establish a set of candidate models that include all possible combinations of six core predictors [27]. The best-fit model was selected from the candidate models based on Akaike’s information criterion (AIC) [56]. The model with the lowest AIC is considered as the best-fit model (S4 Table). In the next step, we established spatial linear models (SLM) [4] for the best models identified by GLM, to avoid inflation of type I errors and invalid parameter estimate owning to spatial autocorrelation [27]. We used simultaneous autoregressive (SAR) models to account for spatial autocorrelation. SAR spatial error models were used because their performance is better than the SAR lag model and SAR mix models when dealing with spatially autocorrelated species distribution data [57]. We examined a range of possible lag distances (100, 200, 400, 600, 800 and 1000 km) for each model and used Moran’ s I coefficient to determine the degree of spatial autocorrelation in the residual of models. Spatial error models with a lag distance of 100 km generally accounted best for the spatial structure in the data set according to the minimum value of AIC. As r^2^ values are not directly provided for SAR models, we used a pseudo-r^2^ value to assess the SLM model goodness fit. Pseudo-r^2^ was calculated as the squared Pearson correlation between predicted and observed (species richness) values [57]. We also identified the contribution of each predictor to the residuals of species richness in the best-fit SLM by testing z value for its significance [27]. Finally, we compared multivariate regressions of six predictors with that of 19 predictors to examine the robustness of six-predictor best-fit GLM and SLM [27].

#### Area-effect Test

Data on the residuals of species richness were used to establish multivariate models, although this may lead to biased parameter estimates [58]. To test the correctness of our methods, we also established multivariate models using raw data and treating area as a variable in the model. We found that there was no obvious difference in multivariate models between the two methods (S5 Table). Therefore, results of residuals of species richness were presented in this study.

#### Sampling-bias Test

The multivariate models can be influenced by geographical sampling bias [59–60]. Two principles were designed to deal with sampling bias. The first principle is that the study area of the target region should remain unchanged, and the second is that sampling units should be randomly selected. To test the impacts of sampling bias on multivariate models, we used stratified random sampling. The study area was divided into 32 strata according to the administrative boundary of provinces, autonomous regions or municipalities in China. Random sampling of counties was conducted in each stratum. We selected 60% of the number of counties for each stratum and obtained a subset of counties, which were randomly selected and minimized sampling bias. We established SLM multivariate models for the residuals of species richness of mammals and resident birds in this subset of counties. We compared the prominent predictors of SLM models for this subset of counties to that of all counties in China in order to test the impact of sampling bias. We found that the prominent predictors were the same after the treatment. Therefore, the residuals of richness data of all counties were used in this study.

## Results

### Patterns of mammal and bird species richness

Both mammals and birds are widely distributed across China. Mammal species richness was higher in the south than in the north, and in the mountains than in the plains. Such regions harbor the highest mammal species richness, i.e. the southeastern Himalaya Mountains, the Hengduan Mountains, the Minshan Mountains, the Qionglai Mountains, the Qinling Mountains, the Daba mountains, the Wuyi Mountains, the Xishuangbanna in Yunnan Province, the border regions of southwestern Guangxi, and the mountains of central and southern part of Hainan. All these hot spots covered 49 assessment units of ten provinces (autonomous regions). Mammal species richness reached the maximum (i.e., 107) in Jingdong, Yunnan. Species richness of mammals ranged from 45 to 69 in such regions, i.e. the Nanling Mountains, the Wushan Mountains, the Dalou Mountains, the mountains in the borders of Zhejiang Province and Anhui Province, the Xiao Hinggan Mountains, the Changbai Mountains, the Helan Mountains, the Qilian Mountains and the Altai Mountains. Species richness in other regions was less than 18, e.g., the North China Plain and the Sichuan Basin.

Resident birds share the similar distribution patterns. Our result showed that resident bird species richness was higher in the south than in the north, and in the mountains than in the plains and plateaus. The highest species richness of resident birds occurs in the southeastern Himalaya Mountains, the Hengduan Mountains, the Daliang Mountains, the Gaoligong Mountains, the Xishuangbanna and the border regions of southwestern Guangxi. The hot spots included 37 assessment units of four provinces in China. The species richness of resident birds reached the maximum (i.e., 323) in Tsonag, Tibet. Species richness of resident birds ranged from 99 to 170 in such regions, i.e. the hills in Guangdong, Taiwanese mountains, the Wuyi Mountains, the Nanling Mountains, the Qinling Mountains, the Daba Mountains, the Dalou Mountains and the Minshan Mountains. The species richness was less than 33 in most assessment units of northwestern China (e.g., the Qinghai-Tibet Plateau, the Inner Mongolian Plateau and the Tarim Basin).

### Determinants of mammal and bird species richness

We identified elevation variability as the most important predictor of species richness of mammals (z = 9.49, P<0.001) and resident birds (z = 9.33, P<0.001) in China (Tables 2 and 3). Mean annual precipitation, net primary productivity and environmental variability were also prominent predictors of species richness of mammals and resident birds. These core predictors together explained 53% and 66% of the variance of species richness of these two taxa, respectively (Tables 2 and 3). When considering all 19 environmental variables, the change in model fit was small (Δr^2^ = 0.01). Therefore, we are confident of the robustness of these best models. Habitat heterogeneity hypothesis explained most of the variance of species richness of mammals and resident birds. We also found broad support for the water-energy hypothesis, the productivity hypothesis and the environmental stability hypothesis (Tables 2 and 3).

**Table 2:** SLM multivariate models for the residuals of species richness of all mammals and its habitat groups.

	Variables		All mammals	Forest mammals	Shrub mammals	Grassland mammals	Desert mammals	Farmland mammals	Cave mammals
Best model with 6 predictors	Mean annual precipitation	z	5.11***					2.39*	
	Precipitation of the wettest quarter	z					-2.47*		
	Precipitation of the driest quarter	z							0.77
	Mean annual dryness	z		5.76***	5.74***				
	Mean annual temperature	z				4.74***	2.40*		
	Maximum temperature of the warmest month	z				-5.69***		2.09*	4.14***
	Annual actual evapotranspiration	z				5.60***	-0.56		
	Net primary productivity	z	3.58***	8.91***	7.46***			3.24**	1.72
	Normalized difference vegetation index	z		1.35	2.21*			2.20*	
	Temperature annual range	z		1.84	-2.11*	7.13***	5.44***		
	Mean diurnal range	z							-1.66
	Precipitation seasonality	z	-2.70**			-3.95***		-2.30*	-0.58
	Elevation variability	z	9.49***	11.76***	11.51***				
	Mean elevation	z					3.56***		
	Main land cover type	z	-6.24***	-6.41***	-5.88***				
	AIC		-1502.3	-1209	-1702	-2119	-2204	-1759	-1124
	Fitted values	r^2^	0.53	0.61	0.64	0.51	0.50	0.47	0.54
	Moran’s I		-0.02	-0.03	-0.01	-0.03	-0.05	-0.02	-0.02
19-predictor model	AIC		-1524.2	-1329	-1768	-2200	-2282	-2002	-1363
	Fitted values	r^2^	0.54	0.64	0.66	0.53	0.52	0.52	0.59

Six variables that explained most of the variance of the residuals of species richness were selected based on univariate regression models and hierarchical partitioning. We established the best multivariate model using multivariable GLM regression. To avoid inflation of type I errors and invalid parameter estimate owning to spatial autocorrelation, we then performed SLM multivariate regression (see Methods). All continuous variables were log10-transformed (n = 2376; *: Pr(>|z|)<0.05; **: Pr(>|z|)<0.01; ***: Pr(>|z|)<0.001).

**Table 3:** SLM multivariate models for the residuals of species richness of all resident birds and its habitat groups.

	Variables		All birds	Forest birds	Shrub birds	Grassland birds	Desert birds	Wetland birds
Best model with 6 predictors	Mean annual precipitation	z	2.76**	4.84***	1.72			1.05
	Precipitation of the driest quarter	z				-5.93***	-5.90***	
	Mean annual temperature	z				3.61***		
	Maximum temperature of the warmest month	z				-4.12***		
	Minimum temperature of the coldest month	z		5.83***	9.03***			0.02
	Annual actual evapotranspiration	z					0.02	0.25
	Net primary productivity	z	1.98*	3.95***	2.58**			
	Normalized difference vegetation index	z					-5.06***	
	Temperature annual range	z	-7.46***					-10.88***
	Mean diurnal range	z					2.92**	
	Temperature seasonality	z				0.28	-3.92***	
	Precipitation seasonality	z						-0.18
	Elevation variability	z	9.33***	11.65***	11.00***			
	Main land cover type	z	-3.29*	-3.73***	-3.47***			-5.68***
	AIC		-810.9	-594	-1297	-1749	-1947	-1253
	Fitted values	r^2^	0.66	0.65	0.63	0.54	0.72	0.77
	Moran’s I		-0.04	0.00	-0.05	-0.06	-0.10	-0.05
19-predictor model	AIC		-875.4	-678	-1344	-1833	-2170	-1555
	Fitted values	r^2^	0.67	0.67	0.64	0.56	0.74	0.80

The details of analysis are the same with that in Table 2 (n = 2376; *: Pr(>|z|)<0.05; **: Pr(>|z|)<0.01; ***: Pr(>|z|)<0.001).

We also established SLM models for species richness of mammals and resident birds in different habitat groups (Tables 2 and 3). The change in fitted values between SLM models with six predictors and that with 19 predictors was small (Δr^2^ ranges from 0.01 to 0.05). Therefore, we are confident of the robustness of these best models. Elevation variability was the most important predictor of species richness of forest and shrub mammals (z = 11.76 and 11.51, respectively, P<0.001) and resident birds (z = 11.65 and 11.00, respectively, P<0.001) (Tables 2 and 3). However, elevation variability was not a prominent predictor of species richness of the habitat groups (i.e., grassland, desert, farmland or cave mammals; grassland, desert or wetland birds). Elevation variability was a relatively independent variable in China, with low correlations with other variables except mean elevation (S1 Table). For example, the correlation coefficients between elevation variability and precipitation, temperature and productivity were less than 0.2. Temperature annual range was the most important predictor of species richness of grassland and desert mammals (z = 7.13 and 5.44, respectively, P<0.001) (Table 2). The maximum temperature of the warmest month was the only prominent predictor of species richness of cave mammals (bats) (z = 4.14, P<0.001) (Table 2). Precipitation of the driest quarter was the most important predictor of species richness of grassland and desert birds (z = -5.93 and -5.90, respectively, P<0.001) (Table 3). Temperature annual range was the most important predictor of species richness of wetland birds (z = -10.88, P<0.001) (Table 3).

## Discussion

### Determinants of overall mammal and bird species richness

We found that elevation variability was the most important predictor of species richness of mammals and resident birds in the terrestrial and inland water ecosystems of China since the higher z values were yielded in the study (z = 9.49 and 9.33 for mammals and resident birds, respectively) (Tables 2 and 3). Similar findings were reported in the previous studies of species richness at the global and regional scales, e.g., mammals in the continental USA and southern Canada [7], birds across the globe [14], birds in South America [8], birds in North America [10] and mammals and birds in Southeast Spain [61]. Regions with high variability in elevation provide diverse habitats, and thus harbor more species.

Some previous findings were inconsistent with the result of this study. Qian [26] reported that energy and water were the most dominant determinants of species richness of all vertebrate groups except reptiles at the global scale, which may be caused by the differences in extent (global scale in Qian’s study [26] vs. national scale in our study) and grain (10056–296019 km^2^ in Qian’s study [26] vs. mean county area of 3908.7 km^2^ in our study). Luo et al. [21] supported the energy hypothesis and the environmental stability hypothesis, with the annual mean temperature as the main factor, followed by annual precipitation and NDVI in the study of environmental effects on vertebrate species richness in China. It may be due to the fact that mammals, birds, reptiles, and amphibians were treated together at a coarser grain in Luo et al.’s [21]. Lin et al. [62] discovered that NDVI was the most important determinant of mammal species richness in China. It may result from the resolution at a coarser grain of 100 × 100 km^2^. Ding et al. [63] considered primary productivity as the key determinant underpinning patterns of bird species richness in East Asia. It is probably due to the smaller extent with higher variability in elevation and the finer grain used in our study. Liu et al. [64] identified annual precipitation and annual mean temperature as the most prominent factors accounting for avian species richness in China. The inconsistency between our analyses and Liu et al. ‘s study [64] may stem from the fact that all bird species were addressed and the coarser grain of 100 × 100 km^2^ was used by Liu et al. [64].

### Determinants of mammal and bird species richness among habitat groups

A unified mechanism associated with environmental determinants can’t often be proposed when explaining species richness [6]. Thus, habitat grouping may be an appropriate approach to advance the study of the species richness patterns. Taking mammals and birds as the pioneers, our study provided insights into group-specific patterns of species richness among the habitat groups. It suggests that there were differences in species richness patterns of mammals and resident birds based on habitat groups and that the role of each hypothesis and their combinations in explaining patterns of species richness also differed among habitat groups. We found that some environmental variables that were not dominant in explaining the overall species richness of mammal and resident birds stood out prominently in the habitat groups, which should receive more attention in the making of conservation policies.

#### Forest and shrub mammals and birds

Our result showed that elevation variability was a prominent determinant of species richness of forest and shrub mammals and birds in China (z = 11.76 and 11.51 for forest and shrub mammals respectively; z = 11.65 and 11.00 for forest and shrub birds respectively) (Tables 2 and 3). Similar results were also reported in previous studies of birds in temperate Danish forests [65], and mammals and block-endemic birds within the Eastern Arc Mountains of Kenya and Tanzania [66]. According to the definition of UNEP-WCMC [67], China harbors approximately 4.6 million km^2^ mountains accounting for 48% of its total land area [12] (Fig 1). Forest and shrub mammals and birds are mainly distributed in the mountainous regions. Compared to other ecosystems, mountainous regions possess the distinct elevation variability, create diverse niches (e.g. the vertical partitioning of resources and nesting sites) for the species formation and specialization and eventually influence the diversity of mammal and bird species richness by vegetation physiognomy [65].

#### Grassland and desert mammals and birds

Temperature annual range was the most important predictor of species richness of grassland and desert mammals instead of elevation variability (z = 7.13 and 5.44 for grassland and desert mammals, respectively, P<0.001) (Table 2). Temperature annual range was negatively and significantly correlated with mean annual precipitation (r = -0.81, P<0.01) and mean annual temperature (r = -0.78, P<0.01). As for grassland and desert birds, precipitation of the driest quarter was the most important predictor of the species richness (z = -5.93 and -5.90 for grassland and desert birds, respectively, P<0.001) (Table 3). Grasslands and deserts are mainly distributed in the arid region of northwestern China and the Qinghai-Tibet Plateau. Both of the regions harbor the low precipitation and temperature: the arid region of northwestern China is characterized by its typical arid and semi-arid climate with mean annual precipitation around 254 mm, and the Qinghai-Tibet Plateau is the world's largest and highest plateau with low temperatures (Mean annual temperature is around 0.1°C). Therefore, the unique climate of low precipitation and temperature greatly affects the distribution of mammal and resident bird species in the two regions. Our conclusion was partially supported by Li et al. [68], who considered that vertebrate richness was more strongly determined by energy availability and supported the energy hypothesis (the “water-energy dynamics” version). In the arid region of northwestern China and the Qinghai-Tibet Plateau, precipitation and/or temperature (ambient energy) greatly influences vegetation growth (e.g., net primary production) and the structure of plant community as the key factors, and thus indirectly affects the species richness of animals since the plants can offer food and habitats for animals [68–71]. The endemic species in both regions have developed unique adaptation to extreme environments, such as draught-and-coldness tolerance. Understanding the special characters and their synergic relationship with climate change may enhance the conservation of the endemic species across China.

#### Cave mammals

The maximum temperature of the warmest month was found to mostly account for species richness of cave mammals (bats) (z = 4.14, P<0.001; Table 2). Our result can be explained by the bats’ physiological features. As the bats lack the ability of thermoregulatory control in the inactive state, their resting temperature and metabolic rate is dependent on the ambient temperature in the environment [72]. In addition, temperature can also have effects on bat species richness indirectly through food resources and availability [73–74]. It was discovered that bats prefer stable caves with the temperature higher than 13°C [75]. Our analysis partially supported the meta-analysis of bat species richness at the global scale [76], which identified temperature and water availability as the major drivers of the bat species richness. The discrepancy between our analysis and previous study may result from the fact that bats are mostly distributed in southern China where relatively sufficient precipitation is not a prominent predictor for species richness of bats.

#### Wetland birds

Temperature annual range was the most important predictor of resident wetland birds in China (z = -10.88; Table 3). Most waterbirds aggregate in relatively small wetland areas [77–78]. In addition, waterbirds do not inhabit the wetlands with low temperature and freezing in winter which prevents the birds from foraging. Resident wetland birds are mainly distributed in southern China with a warm and stable climate. By contrast, fewer resident wetland birds inhabit northern China where temperature is low and shows large variability [42]. Thus, a stable habitat with suitable temperatures contributes dominantly to higher species richness of wetland birds.

In addition, main land cover type was the second important predictor of species richness of wetland birds (z = -5.68, P<0.001; Table 3). This is because wetland birds depend highly on wetlands and accordingly main land cover type plays an important role in the distribution of wetland birds. Similar findings were reported in the studies of waterbirds in Chongming Dongtan of the Yangtze River Estuary [79], and the Sanjiang Plain, China [80]. Some studies have shown that the bodies of wetlands, including large rivers (e.g., the Yangtze River, and the Yellow River) and major freshwater lakes (e.g., the Dongting Lake), have been strongly affected by human-induced land use changes at an alarming rate in China [81], which further posed a threat to bird species richness. The similar effect of land cover types on wetland bird species richness was discovered in Ontario, Canada [82] and the Conterminous United States [83]. Thus, conservation policies should consider the land use change as an indispensible factor to protect wetland birds.

### Hypotheses explaining mammal and bird species richness

Although we found support for the importance of energy, habitat heterogeneity and environmental stability hypotheses, their relative contributions to explaining patterns of species richness varied strongly among habitat groups. The sets of prominent predictors of species richness in habitat groups were generally diverse. The habitat heterogeneity hypothesis was one of the two most important hypotheses for species richness of mammals, resident birds, forest and shrub mammals and resident birds. The environmental stability hypothesis played the most important role in explaining variation of species richness of wetland birds, and the water-energy hypothesis played the most important role for desert birds. The ambient energy (temperature) hypothesis was the most important hypothesis for species richness of cave mammals. The synergistic effects of specialization and adaption to different habitats likely lead to differences in species richness patterns of habitat groups. Therefore, understanding the richness gradients requires the appropriate de-composition of constituent groups.

## Conclusions and Future Work

In summary, our analyses revealed a substantial divergence in geographical richness patterns among habitat groups and highlighted the group-specific and disparate environmental associations that underpin them. Further studies are needed to identify habitat groups of animal species based on different purposes. Furthermore, our results clearly demonstrated how large-scale patterns of species richness were best viewed as the overlaid response of different groups to diverse environmental factors [6, 84]. Understanding of the patterns, status and trends of biodiversity requires the identification and inclusion of different habitat groups. Our findings suggest that biodiversity surveys and monitoring will benefit from addressing many representative habitat groups. As we demonstrate, a focus on overall species richness alone might lead to incomplete or misguided understanding of spatial patterns. Conservation policies and prioritization strategies that consider a broad spectrum of habitat groups from multiple taxa will be more successful in safeguarding the multiple services of biodiversity.

## Supporting Information

S1 Appendix Checklist of mammals based on habitat groups. (XLS)

S2 Appendix Checklist of resident birds based on habitat groups. (XLS)

S3 Appendix Residuals of species richness of mammals and resident birds and their habitat groups. (XLS)

S1 Fig Flow diagram of the statistical methods. (DOC)

S1 Table Correlations between environmental variables. Number in the first row corresponds to the number of variables in the first column. 1-Mean annual precipitation. Spearman (two-sided) correlation was performed (n = 2376; **, P<0.01; *P<0.05).
(DOC)

S2 Table Variables selected based on Spearman (two-sided) correlation analysis and univariate regression for species richness of mammals and resident birds and their habitat groups. Spearman (two-sided) correlation analysis between any two variables in each category was performed. The deviance of each variable in each category explaining species richness was calculated using univariate regression models. If the correlation coefficient between two variables in a category was >0.7, we removed strongly intercorrelated variables and retained the variables that explained more deviance in univariate regression models.
(DOCX)

S3 Table Top six predictors selected based on hierarchical partitioning analysis. *P<0.05.
(DOCX)

S4 Table GLM multivariate models for the residuals of species richness of mammals and resident birds and their habitat groups. Six variables that explained most of the variance of the residuals of species richness were selected based on univariate regression models and hierarchical partitioning. The results of GLM multivariable regression based on six variables were listed with that based on 19 variables as a comparison.
(DOCX)

S5 Table SLM multivariate models for species richness of mammals and birds and treating area as a variable in the models. Six variables that explained most of the variance of species richness were selected based on univariate regression models and hierarchical partitioning. We established the best multivariate model using GLM multivariable regression. To avoid inflation of type I errors and invalid parameter estimate owning to spatial autocorrelation, we then performed SLM multivariate regression (see Methods). Species richness and all continuous variables were log10-transformed (n = 2376; *: Pr(>|z|)<0.05; **: Pr(>|z|)<0.01; ***: Pr(>|z|)<0.001).
(DOCX)
