# Supplementary material for: Determinants of Mammal and Bird Species Richness in China Based on Habitat Groups
Source: PLoS One. 2015 Dec 2;10(12):e0143996. doi: 10.1371/journal.pone.0143996 (PMC4668080; doi:10.1371/journal.pone.0143996)
Supplement: S1 Fig — (DOC) [file pone.0143996.s004.doc]

**S1 Fig. Flow diagram of the statistical methods**

Residuals of species richness

Predictor variables (five hypotheses)

Univariate models

（greater deviances）

Spearman correlation analysis

(correlation coefficient >0.7)

Hierarchical partitioning

Log10-transformed and area effects removed

Select predictors for each hypothesis

Six core predictors that have the most independent effects on the residuals of species richness

Generalized linear models (GLM)

(the smallest AIC value)

Best spatial linear models (SLM)

(AIC value, significant test)

**Test the hypotheses and identify the main predictors**

**Data processing**

**Variable selection**

A set of candidate models

The best GLM model

SAR spatial error models to avoid spatial autocorrelation (lag distance; Moran’s I)

**Model selection**
