# Supplementary material for: Determinants of Mammal and Bird Species Richness in China Based on Habitat Groups
Source: PLoS One. 2015 Dec 2;10(12):e0143996. doi: 10.1371/journal.pone.0143996 (PMC4668080; doi:10.1371/journal.pone.0143996)
Supplement: S1 Table — Number in the first row corresponds to the number of variables in the first column. 1-Mean annual precipitation. Spearman (two-sided) correlation was performed (n = 2376; **, P<0.01; *P<0.05). (DOC) [file pone.0143996.s005.doc]

**S1 Table. Correlations between environmental variables.**

| **Variables** | 1 | 2 | 3 | 4 | 5 | 6 | 7 | 8 | 9 | 10 | 11 | 12 | 13 | 14 | 15 | 16 | 17 | 18 |
| --- | --- | --- | --- | --- | --- | --- | --- | --- | --- | --- | --- | --- | --- | --- | --- | --- | --- | --- |
| 2. Precipitation of the wettest quarter | 0.96^**^ |  |  |  |  |  |  |  |  |  |  |  |  |  |  |  |  |  |
| 3. Precipitation of the driest quarter | 0.92^**^ | 0.83^**^ |  |  |  |  |  |  |  |  |  |  |  |  |  |  |  |  |
| 4. Mean annual dryness | 0.96^**^ | 0.91^**^ | 0.88^**^ |  |  |  |  |  |  |  |  |  |  |  |  |  |  |  |
| 5. Mean annual temperature | 0.85^**^ | 0.82^**^ | 0.83^**^ | 0.72^**^ |  |  |  |  |  |  |  |  |  |  |  |  |  |  |
| 6. Maximum temperature of the warmest month | 0.43^**^ | 0.36^**^ | 0.57^**^ | 0.29^**^ | 0.69^**^ |  |  |  |  |  |  |  |  |  |  |  |  |  |
| 7. Minimum temperature of the coldest month | 0.87^**^ | 0.85^**^ | 0.81^**^ | 0.76^**^ | 0.97^**^ | 0.53^**^ |  |  |  |  |  |  |  |  |  |  |  |  |
| 8. Annual potential evapotranspiration | 0.53^**^ | 0.55^**^ | 0.51^**^ | 0.31^**^ | 0.79^**^ | 0.70^**^ | 0.72^**^ |  |  |  |  |  |  |  |  |  |  |  |
| 9. Annual actual evapotranspiration | 0.92^**^ | 0.88^**^ | 0.86^**^ | 0.86^**^ | 0.83^**^ | 0.44^**^ | 0.85^**^ | 0.56^**^ |  |  |  |  |  |  |  |  |  |  |
| 10. Net primary productivity | 0.83^**^ | 0.84^**^ | 0.72^**^ | 0.78^**^ | 0.73^**^ | 0.22^**^ | 0.80^**^ | 0.49^**^ | 0.78^**^ |  |  |  |  |  |  |  |  |  |
| 11.Normalized difference vegetation index | 0.46^**^ | 0.43^**^ | 0.42^**^ | 0.51^**^ | 0.21^**^ | 0.15^**^ | 0.19^**^ | 0.03 | 0.37^**^ | 0.44^**^ |  |  |  |  |  |  |  |  |
| 12.Mean diurnal range | -0.82^**^ | -0.73^**^ | -0.83^**^ | -0.78^**^ | -0.80^**^ | -0.51^**^ | -0.83^**^ | -0.36^**^ | -0.74^**^ | -0.67^**^ | -0.32^**^ |  |  |  |  |  |  |  |
| 13.Temperature seasonality | -0.70^**^ | -0.70^**^ | -0.54^**^ | -0.66^**^ | -0.64^**^ | -0.02 | -0.78^**^ | -0.42^**^ | -0.70^**^ | -0.72^**^ | -0.03 | 0.52^**^ |  |  |  |  |  |  |
| 14.Temperature annual range | -0.81^**^ | -0.80^**^ | -0.68^**^ | -0.76^**^ | -0.78^**^ | -0.18^**^ | -0.90^**^ | -0.51^**^ | -0.80^**^ | -0.80^**^ | -0.10^**^ | 0.71^**^ | 0.96^**^ |  |  |  |  |  |
| 15.Precipitation seasonality | -0.70^**^ | -0.53^**^ | -0.82^**^ | -0.70^**^ | -0.64^**^ | -0.44^**^ | -0.64^**^ | -0.32^**^ | -0.67^**^ | -0.52^**^ | -0.28^**^ | 0.70^**^ | 0.45^**^ | 0.55^**^ |  |  |  |  |
| 16.Elevation variability | 0.05^*^ | 0.06^**^ | -0.08^**^ | 0.13^**^ | -0.17^**^ | -0.54^**^ | -0.03 | -0.19^**^ | 0.03 | 0.14^**^ | 0.03 | 0.18^**^ | -0.38^**^ | -0.24^**^ | -0.11^**^ |  |  |  |
| 17.Mean elevation | -0.27^**^ | -0.24^**^ | -0.41^**^ | -0.18^**^ | -0.43^**^ | -0.74^**^ | -0.26^**^ | -0.34^**^ | -0.24^**^ | -0.07^**^ | -0.18^**^ | 0.44^**^ | -0.24^**^ | -0.04^*^ | 0.15^**^ | 0.79^**^ |  |  |
| 18.Main land cover type | -0.38^**^ | -0.35^**^ | -0.30^**^ | -0.44^**^ | -0.16^**^ | 0.11^**^ | -0.23^**^ | -0.04^*^ | -0.31^**^ | -0.36^**^ | -0.35^**^ | 0.17^**^ | 0.33^**^ | 0.30^**^ | 0.33^**^ | -0.41^**^ | -0.27^**^ |  |
| 19.Number of land cover types | -0.15^**^ | -0.12^**^ | -0.20^**^ | -0.08^**^ | -0.24^**^ | -0.38^**^ | -0.20^**^ | -0.24^**^ | -0.16^**^ | -0.13^**^ | -0.21^**^ | 0.27^**^ | -0.02 | 0.06^**^ | 0.06^**^ | 0.48^**^ | 0.38^**^ | -0.14^**^ |

Number in the first row corresponds to the number of variables in the first column. 1-Mean annual precipitation. Spearman (two-sided) correlation was performed (n=2376; **, P<0.01; *P<0.05).
