# Supplementary material for: Determinants of Mammal and Bird Species Richness in China Based on Habitat Groups
Source: PLoS One. 2015 Dec 2;10(12):e0143996. doi: 10.1371/journal.pone.0143996 (PMC4668080; doi:10.1371/journal.pone.0143996)
Supplement: S2 Table — Spearman (two-sided) correlation analysis between any two variables in each category was performed. The deviance of each variable in each category explaining species richness was calculated using univariate regression models. If the correlation coefficient between two variables in a category was >0.7, we removed strongly intercorrelated variables and retained the variables that explained more deviance in univariate regression models. (DOCX) [file pone.0143996.s006.docx]

**S2 Table. Variables selected based on Spearman (two-sided) correlation analysis and univariate regression for species richness of mammals and resident birds and their habitat groups.**

| **Variables** | **Deviance (%)** |
| --- | --- |
| **All mammals** |  |
| Mean annual precipitation | 16.4 |
| Maximum temperature of the warmest month | 0.1 |
| Minimum temperature of the coldest month | 9.4 |
| Net primary productivity | 17.2 |
| Temperature annual range | 12.4 |
| Precipitation seasonality | 2.1 |
| Elevation variability | 10.4 |
| Main land cover type | 6.4 |
| Number of land cover types | 1.1 |
| **Forest mammals** |  |
| Mean annual dryness | 23.2 |
| Maximum temperature of the warmest month | 0.2 |
| Minimum temperature of the coldest month | 7.0 |
| Net primary productivity | 26.4 |
| Normalized difference vegetation index | 0.6 |
| Temperature annual range | 9.8 |
| Precipitation seasonality | 1.5 |
| Elevation variability | 10.5 |
| Main land cover type | 6.9 |
| Number of land cover types | 2.3 |
| **Shrub mammals** |  |
| Mean annual dryness | 34.2 |
| Maximum temperature of the warmest month | 0.5 |
| Minimum temperature of the coldest month | 14.7 |
| Net primary productivity | 31.6 |
| Normalized difference vegetation index | 0.1 |
| Temperature annual range | 19.9 |
| Precipitation seasonality | 2.7 |
| Elevation variability | 11.6 |
| Main land cover type | 9.3 |
| Number of land cover types | 2.7 |
| **Grassland mammals** |  |
| Precipitation of the driest quarter | 23.1 |
| Mean annual temperature | 25.3 |
| Maximum temperature of the warmest month | 2.4 |
| Annual actual evapotranspiration | 10.0 |
| Normalized difference vegetation index | 4.8 |
| Temperature annual range | 23.9 |
| Precipitation seasonality | 0.1 |
| Mean elevation | 1.6 |
| Main land cover type | 1.2 |
| **Desert mammals** |  |
| Precipitation of the wettest quarter | 22.2 |
| Mean annual temperature | 13.0 |
| Maximum temperature of the warmest month | 4.1 |
| Annual actual evapotranspiration | 20.5 |
| Normalized difference vegetation index | 0.3 |
| Temperature annual range | 17.0 |
| Mean elevation | 3.7 |
| Main land cover type | 4.5 |
| **Farmland mammals** |  |
| Mean annual precipitation | 8.1 |
| Maximum temperature of the warmest month | 4.8 |
| Minimum temperature of the coldest month | 0.8 |
| Net primary productivity | 10.4 |
| Normalized difference vegetation index | 1.2 |
| Mean diurnal range | 6.6 |
| Temperature seasonality | 0.5 |
| Precipitation seasonality | 0.4 |
| Elevation variability | 1.0 |
| Main land cover type | 1.3 |
| Number of land cover types | 0.4 |
| **Cave mammals** |  |
| Precipitation of the driest quarter | 13.2 |
| Mean annual temperature | 13.6 |
| Maximum temperature of the warmest month | 1.0 |
| Net primary productivity | 8.1 |
| Mean diurnal range | 10.6 |
| Precipitation seasonality | 0.9 |
| Elevation variability | 1.4 |
| Main land cover type | 4.6 |
| Number of land cover types | 0.5 |
|  |  |
| **All resident birds** |  |
| Mean annual precipitation | 25.4 |
| Maximum temperature of the warmest month | 0.3 |
| Minimum temperature of the coldest month | 24.4 |
| Net primary productivity | 17.1 |
| Normalized difference vegetation index | 0.3 |
| Temperature annual range | 29.2 |
| Precipitation seasonality | 0.1 |
| Elevation variability | 10 |
| Mean elevation | 4.6 |
| Main land cover type | 4.9 |
| Number of land cover types | 1.6 |
| **Forest birds** |  |
| Mean annual precipitation | 27.6 |
| Maximum temperature of the warmest month | 1.6 |
| Minimum temperature of the coldest month | 17.2 |
| Net primary productivity | 22.1 |
| Temperature annual range | 22.8 |
| Precipitation seasonality | 0.2 |
| Elevation variability | 8.1 |
| Main land cover type | 5.1 |
| Number of land cover types | 1.5 |
| **Shrub birds** |  |
| Mean annual precipitation | 23.7 |
| Maximum temperature of the warmest month | 2.8 |
| Minimum temperature of the coldest month | 19.0 |
| Net primary productivity | 16.2 |
| Normalized difference vegetation index | 0.4 |
| Temperature annual range | 25.0 |
| Precipitation seasonality | 1.4 |
| Elevation variability | 9.4 |
| Main land cover type | 5.4 |
| Number of land cover types | 0.8 |
| **Grassland birds** |  |
| Precipitation of the driest quarter | 5.9 |
| Mean annual temperature | 5.6 |
| Maximum temperature of the warmest month | 14.5 |
| Net primary productivity | 1.3 |
| Normalized difference vegetation index | 0.5 |
| Mean diurnal range | 4.6 |
| Temperature seasonality | 8.4 |
| Mean elevation | 8.3 |
| **Desert birds** |  |
| Precipitation of the driest quarter | 40.4 |
| Mean annual temperature | 27.1 |
| Annual actual evapotranspiration | 31.3 |
| Normalized difference vegetation index | 0.2 |
| Mean diurnal range | 35.9 |
| Temperature seasonality | 0.9 |
| Mean elevation | 10.2 |
| Main land cover type | 6.4 |
| **Wetland birds** |  |
| Mean annual precipitation | 42.7 |
| Maximum temperature of the warmest month | 3.8 |
| Minimum temperature of the coldest month | 47.6 |
| Annual actual evapotranspiration | 30.7 |
| Normalized difference vegetation index | 1.0 |
| Temperature annual range | 55.5 |
| Precipitation seasonality | 2.1 |
| Elevation variability | 5.5 |
| Main land cover type | 8.3 |
| Number of land cover types | 0.9 |

Spearman (two-sided) correlation analysis between any two variables in each category was performed. The deviance of each variable in each category explaining species richness was calculated using univariate regression models. If the correlation coefficient between two variables in a category was >0.7, we removed strongly intercorrelated variables and retained the variables that explained more deviance in univariate regression models.
