# Supplementary material for: Determinants of Mammal and Bird Species Richness in China Based on Habitat Groups
Source: PLoS One. 2015 Dec 2;10(12):e0143996. doi: 10.1371/journal.pone.0143996 (PMC4668080; doi:10.1371/journal.pone.0143996)
Supplement: S3 Table — *P<0.05. (DOCX) [file pone.0143996.s007.docx]

**S3 Table. Top six predictors selected based on hierarchical partitioning analysis.**

| **No.** | | **Variables** | **I.perc** | **Z.score** | **sig95** |
| --- | --- | --- | --- | --- | --- |
|  | | **All mammals** |  |  |  |
| 1 | | Net primary productivity | 21.20924 | 157 | * |
| 2 | | Mean annual precipitation | 17.31435 | 143.4 | * |
| 3 | | Elevation variability | 16.80187 | 133.23 | * |
| 4 | | Main land cover type | 15.58418 | 111.11 | * |
| 5 | | Temperature annual range | 10.33098 | 74.2 | * |
| 6 | | Precipitation seasonality | 7.17072 | 51.75 | * |
|  | | **Forest mammals** |  |  |  |
| 1 | | Net primary productivity | 24.43922 | 284.39 | * |
| 2 | | Mean annual dryness | 16.81381 | 200.13 | * |
| 3 | | Normalized difference vegetation index | 14.10207 | 145.54 | * |
| 4 | | Elevation variability | 12.97313 | 142.44 | * |
| 5 | | Main land cover type | 11.70277 | 132.21 | * |
| 6 | | Temperature annual range | 6.509491 | 71.36 | * |
|  | | **Shrub mammals** |  |  |  |
| 1 | | Net primary productivity | 20.49944 | 320.92 | * |
| 2 | | Mean annual dryness | 19.36806 | 301.48 | * |
| 3 | | Main land cover type | 10.88691 | 156.83 | * |
| 4 | | Temperature annual range | 10.51703 | 154.39 | * |
| 5 | | Elevation variability | 10.48081 | 151.67 | * |
| 6 | | Normalized difference vegetation index | 9.86253 | 132.6 | * |
|  | | **Grassland mammals** |  |  |  |
| 1 | | Mean annual temperature | 30.04912 | 247.16 | * |
| 2 | | Temperature annual range | 29.30484 | 239.4 | * |
| 3 | | Precipitation of the driest quarter | 12.50282 | 96.07 | * |
| 4 | | Maximum temperature of the warmest month | 9.105329 | 78.14 | * |
| 5 | | Annual actual evapotranspiration | 7.907765 | 64.35 | * |
| 6 | | Precipitation seasonality | 4.813685 | 36.56 | * |
|  | | **Desert mammals** |  |  |  |
| 1 | | Precipitation of the wettest quarter | 26.94924 | 133.81 | * |
| 2 | | Annual actual evapotranspiration | 22.80582 | 121.18 | * |
| 3 | | Temperature annual range | 20.25663 | 93.24 | * |
| 4 | | Mean annual temperature | 12.95327 | 63.03 | * |
| 5 | | Mean elevation | 8.00064 | 35.12 | * |
| 6 | | Normalized difference vegetation index | 3.673244 | 16.35 | * |
|  | | **Farmland mammals** |  |  |  |
| 1 | | Net primary productivity | 17.26981 | 109.47 | * |
| 2 | | Normalized difference vegetation index | 15.17063 | 95.6 | * |
| 3 | | Mean annual precipitation | 10.33462 | 71.73 | * |
| 4 | | Mean diurnal range | 10.2039 | 65.48 | * |
| 5 | | Maximum temperature of the warmest month | 9.660066 | 63.01 | * |
| 6 | | Precipitation seasonality | 8.068637 | 55.94 | * |
|  | | **Cave mammals** |  |  |  |
| 1 | | Maximum temperature of the warmest month | 22.24364 | 122.21 | * |
| 2 | | Mean annual temperature | 17.85328 | 100.25 | * |
| 3 | | Precipitation of the driest quarter | 16.13612 | 93.71 | * |
| 4 | | Mean diurnal range | 11.04369 | 60.23 | * |
| 5 | | Net primary productivity | 9.801653 | 55.85 | * |
| 6 | | Precipitation seasonality | 8.972536 | 41.3 | * |
|  | |  |  |  |  |
|  | | **All resident birds** |  |  |  |
| 1 | | Temperature annual range | 19.60854 | 324.6 | * |
| 2 | | Mean annual precipitation | 14.1649 | 228.27 | * |
| 3 | | Minimum temperature of the coldest month | 13.54289 | 215.91 | * |
| 4 | | Net primary productivity | 10.99145 | 180.69 | * |
| 5 | | Elevation variability | 9.784437 | 143.06 | * |
| 6 | | Main land cover type | 8.585446 | 120.49 | * |
|  | | **Forest birds** |  |  |  |
| 1 | | Mean annual precipitation | 24.9556 | 236.44 | * |
| 2 | | Temperature annual range | 17.11098 | 178.04 | * |
| 3 | | Net primary productivity | 18.48051 | 167.73 | * |
| 4 | | Minimum temperature of the coldest month | 12.51796 | 116.4 | * |
| 5 | | Elevation variability | 10.89979 | 109 | * |
| 6 | | Main land cover type | 7.690696 | 74.01 | * |
|  | **Shrub birds** | |  |  |  |
| 1 | Temperature annual range | | 18.18686 | 248.85 | * |
| 2 | Minimum temperature of the coldest month | | 15.14116 | 188.96 | * |
| 3 | Mean annual precipitation | | 14.75085 | 181.12 | * |
| 4 | Elevation variability | | 11.39183 | 151.39 | * |
| 5 | Net primary productivity | | 10.57188 | 119.61 | * |
| 6 | Main land cover type | | 8.871593 | 116.39 | * |
|  | **Grassland birds** | |  |  |  |
| 1 | Maximum temperature of the warmest month | | 40.42025 | 161.34 | * |
| 2 | Temperature seasonality | | 16.95005 | 69.69 | * |
| 3 | Mean annual temperature | | 13.03664 | 54.6 | * |
| 4 | Mean elevation | | 9.942962 | 40.48 | * |
| 5 | Precipitation of the driest quarter | | 10.72744 | 37.77 | * |
| 6 | Mean diurnal range | | 5.297199 | 21.52 | * |
|  | **Desert birds** | |  |  |  |
| 1 | Precipitation of the driest quarter | | 29.2238 | 241.32 | * |
| 2 | Mean diurnal range | | 22.43556 | 200.43 | * |
| 3 | Annual actual evapotranspiration | | 17.6816 | 151.86 | * |
| 4 | Mean annual temperature | | 13.16211 | 121.54 | * |
| 5 | Normalized difference vegetation index | | 6.580509 | 51.72 | * |
| 6 | Temperature seasonality | | 5.49717 | 45.87 | * |
|  | **Wetland birds** | |  |  |  |
| 1 | Temperature annual range | | 24.79779 | 686 | * |
| 2 | Minimum temperature of the coldest month | | 20.03244 | 532.09 | * |
| 3 | Mean annual precipitation | | 14.76529 | 407.11 | * |
| 4 | Annual actual evapotranspiration | | 10.81234 | 271.89 | * |
| 5 | Precipitation seasonality | | 7.948768 | 206.92 | * |
| 6 | Main land cover type | | 5.37746 | 138.59 | * |

*P<0.05
