# Supplementary material for: Determinants of Mammal and Bird Species Richness in China Based on Habitat Groups
Source: PLoS One. 2015 Dec 2;10(12):e0143996. doi: 10.1371/journal.pone.0143996 (PMC4668080; doi:10.1371/journal.pone.0143996)
Supplement: S4 Table — Six variables that explained most of the variance of the residuals of species richness were selected based on univariate regression models and hierarchical partitioning. The results of GLM multivariable regression based on six variables were listed with that based on 19 variables as a comparison. (DOCX) [file pone.0143996.s008.docx]

**S4 Table. GLM multivariate models for the residuals of species richness of all mammals and resident birds and their habitat groups.**

**S4-1Table. All mammals.**

|  | **Variables** | **Coeff.** | **Std Coeff** | **t value** |
| --- | --- | --- | --- | --- |
| Best model with 6 predictors | Net primary productivity | 0.189 | 0.287 | 9.191 |
|  | Mean annual precipitation | 0.064 | 0.076 | 2.136 |
|  | Elevation variability | 0.103 | 0.252 | 12.87 |
|  | Main land cover type | -0.004 | -0.129 | -6.249 |
|  | Precipitation seasonality | -0.155 | -0.073 | -3.387 |
|  | r^2^ | 0.299 |  |  |
|  | AIC | -774.67 |  |  |
| 19-predictor model | r^2^ | 0.319 |  |  |
|  | AIC | -813.614 |  |  |

**S4-2 Table. Forest mammals.**

|  | **Variables** | **Coeff.** | **Std Coeff** | **t value** |
| --- | --- | --- | --- | --- |
| Best model with 6 predictors | Net primary productivity | 0.291 | 0.378 | 12.306 |
|  | Mean annual dryness | 0.12 | 0.11 | 3.654 |
|  | Normalized difference vegetation index | 0.127 | 0.094 | 3.857 |
|  | Elevation variability | 0.14 | 0.291 | 15.823 |
|  | Main land cover type | -0.004 | -0.105 | -5.46 |
|  | Temperature annual range | 0.473 | 0.073 | 2.956 |
|  | r^2^ | 0.396 |  |  |
|  | AIC | -376.568 |  |  |
| 19-predictor model | r^2^ | 0.432 |  |  |
|  | AIC | -493.972 |  |  |

**S4-3. Table Shrub mammals.**

|  | **Variables** | **Coeff.** | **Std Coeff.** | **t value** |
| --- | --- | --- | --- | --- |
| Best model with 6 predictors | Net primary productivity | 0.206 | 0.284 | 9.927 |
|  | Mean annual dryness | 0.228 | 0.221 | 7.913 |
|  | Main land cover type | -0.004 | -0.111 | -6.19 |
|  | Temperature annual range | -0.29 | -0.047 | -2.06 |
|  | Elevation variability | 0.12 | 0.265 | 15.515 |
|  | Normalized difference vegetation index | 0.096 | 0.075 | 3.306 |
|  | r^2^ | 0.477 |  |  |
|  | AIC | -992.951 |  |  |
| 19-predictor model | r^2^ | 0.501 |  |  |
|  | AIC | -1078.553 |  |  |

**S4-4 Table. Grassland mammals.**

|  | **Variables** | **Coeff.** | **Std Coeff.** | **t value** |
| --- | --- | --- | --- | --- |
| Best model with 6 predictors | Mean annual temperature | 3.849 | 0.817 | 4.894 |
|  | Temperature annual range | 5.228 | 1.081 | 9.622 |
|  | Maximum temperature of the warmest month | -6.881 | -0.856 | -7.144 |
|  | Annual actual evapotranspiration | 0.059 | 0.067 | 2.898 |
|  | Precipitation seasonality | -0.247 | -0.134 | -5.587 |
|  | r^2^ | 0.306 |  |  |
|  | AIC | -1473.443 |  |  |
| 19-predictor model | r^2^ | 0.389 |  |  |
|  | AIC | -1746.146 |  |  |

**S4-5 Table. Desert mammals.**

|  | **Variables** | **Coeff.** | **Std Coeff.** | **t value** |
| --- | --- | --- | --- | --- |
| Best model with 6 predictors | Precipitation of the wettest quarter | -0.17 | -0.201 | -5.07 |
|  | Annual actual evapotranspiration | -0.11 | -0.129 | -3.391 |
|  | Temperature annual range | 1.5 | 0.32 | 9.489 |
|  | Mean annual temperature | 0.739 | 0.162 | 4.725 |
|  | Mean elevation | 0.053 | 0.19 | 7.88 |
|  | r^2^ | 0.263 |  |  |
|  | AIC | -1479.045 |  |  |
| 19-predictor model | r^2^ | 0.352 |  |  |
|  | AIC | -1752.919 |  |  |

**S4-6 Table. Farmland mammals.**

|  | **Variables** | **Coeff.** | **Std Coeff.** | **t value** |
| --- | --- | --- | --- | --- |
| Best model with 6 predictors | Net primary productivity | 0.122 | 0.209 | 5.508 |
|  | Normalized difference vegetation index | 0.162 | 0.158 | 6.201 |
|  | Mean annual precipitation | -0.061 | -0.081 | -2.101 |
|  | Maximum temperature of the warmest month | 0.82 | 0.1 | 4.711 |
|  | Precipitation seasonality | -0.248 | -0.131 | -5.382 |
|  | r^2^ | 0.143 |  |  |
|  | AIC | -879.632 |  |  |
| 19-predictor model | r^2^ | 0.23 |  |  |
|  | AIC | -1102.329 |  |  |

|  | **Variables** | **Coeff.** | **Std Coeff.** | **t value** |
| --- | --- | --- | --- | --- |
| Best model with 6 predictors | Maximum temperature of the warmest month | 2.309 | 0.231 | 10.026 |
|  | Precipitation of the driest quarter | 0.128 | 0.175 | 3.529 |
|  | Mean diurnal range | 1.081 | 0.07 | 1.89 |
|  | Net primary productivity | 0.092 | 0.13 | 5.087 |
|  | Precipitation seasonality | -0.136 | -0.059 | -1.624 |
|  | r^2^ | 0.175 |  |  |
|  | AIC | -15.232 |  |  |
| 19-predictor model | r^2^ | 0.274 |  |  |
|  | AIC | -289.638 |  |  |

**S4-7 Table. Cave mammals.**

**S4-8 Table. All resident birds.**

|  | **Variables** | **Coeff.** | **Std Coeff.** | **t value** |
| --- | --- | --- | --- | --- |
| Best model with 6 predictors | Temperature annual range | -2.084 | -0.279 | -11.54 |
|  | Mean annual precipitation | 0.267 | 0.234 | 6.687 |
|  | Net primary productivity | 0.045 | 0.051 | 1.764 |
|  | Elevation variability | 0.123 | 0.223 | 11.792 |
|  | Main land cover type | -0.002 | -0.062 | -3.169 |
|  | r^2^ | 0.381 |  |  |
|  | AIC | 330.69 |  |  |
| 19-predictor model | r^2^ | 0.415 |  |  |
|  | AIC | 225.223 |  |  |

**S4-9 Table. Forest birds.**

|  | **Variables** | **Coeff.** | **Std Coeff.** | **t value** |
| --- | --- | --- | --- | --- |
| Best model with 6 predictors | Mean annual precipitation | 0.316 | 0.266 | 7.51 |
|  | Net primary productivity | 0.141 | 0.154 | 5.3 |
|  | Minimum temperature of the coldest month | 0.433 | 0.144 | 6.046 |
|  | Elevation variability | 0.148 | 0.258 | 13.998 |
|  | Main land cover type | -0.002 | -0.058 | -2.932 |
|  | r^2^ | 0.365 |  |  |
|  | AIC | 580.093 |  |  |
| 19-predictor model | r^2^ | 0.413 |  |  |
|  | AIC | 422.883 |  |  |

**S4-10 Table. Shrub birds.**

|  | **Variables** | **Coeff.** | **Std Coeff.** | **t value** |
| --- | --- | --- | --- | --- |
| Best model with 6 predictors | Minimum temperature of the coldest month | 0.733 | 0.29 | 12.158 |
|  | Mean annual precipitation | 0.187 | 0.188 | 5.284 |
|  | Elevation variability | 0.13 | 0.269 | 14.565 |
|  | Net primary productivity | 0.043 | 0.056 | 1.925 |
|  | Main land cover type | -0.004 | -0.104 | -5.207 |
|  | r^2^ | 0.364 |  |  |
|  | AIC | -236.582 |  |  |
| 19-predictor model | r^2^ | 0.405 |  |  |
|  | AIC | -363.817 |  |  |

**S4-11 Table. Grassland birds.**

|  | **Variables** | **Coeff.** | **Std Coeff.** | **t value** |
| --- | --- | --- | --- | --- |
| Best model with 6 predictors | Maximum temperature of the warmest month | -7.953 | -0.903 | -9.024 |
|  | Temperature seasonality | 0.408 | 0.229 | 2.966 |
|  | Mean annual temperature | 3.887 | 0.753 | 6.099 |
|  | Precipitation of the driest quarter | -0.147 | -0.228 | -8.276 |
|  | r^2^ | 0.224 |  |  |
|  | AIC | -775.897 |  |  |
| 19-predictor model | r^2^ | 0.263 |  |  |
|  | AIC | -865.434 |  |  |

**S4-12 Table. Desert birds.**

|  | **Variables** | **Coeff.** | **Std Coeff.** | **t value** |
| --- | --- | --- | --- | --- |
| Best model with 6 predictors | Precipitation of the driest quarter | -0.268 | -0.342 | -11.111 |
|  | Mean diurnal range | 3.086 | 0.185 | 6.475 |
|  | Annual actual evapotranspiration | -0.141 | -0.12 | -4.473 |
|  | Normalized difference vegetation index | -0.128 | -0.095 | -4.969 |
|  | Temperature seasonality | 0.095 | 0.044 | 2.267 |
|  | r^2^ | 0.442 |  |  |
|  | AIC | -614.372 |  |  |
| 19-predictor model | r^2^ | 0.622 |  |  |
|  | AIC | -1507.884 |  |  |

**S4-13 Table. Wetland birds.**

|  | **Variables** | **Coeff.** | **Std Coeff.** | **t value** |
| --- | --- | --- | --- | --- |
| Best model with 6 predictors | Temperature annual range | -3.812 | -0.453 | -15.343 |
|  | Minimum temperature of the coldest month | 0.379 | 0.117 | 3.721 |
|  | Mean annual precipitation | 0.275 | 0.215 | 6.156 |
|  | Annual actual evapotranspiration | -0.086 | -0.056 | -1.905 |
|  | Precipitation seasonality | -0.221 | -0.069 | -4.031 |
|  | Main land cover type | -0.006 | -0.125 | -8.104 |
|  | r^2^ | 0.605 |  |  |
|  | AIC | -180.601 |  |  |
| 19-predictor model | r^2^ | 0.654 |  |  |
|  | AIC | -463.884 |  |  |

Six variables that explained most of the variance of the residuals of species richness were selected based on univariate regression models and hierarchical partitioning. The results of GLM multivariable regression based on six variables were listed with that based on 19 variables as a comparison.
