# Supplementary material for: Determinants of Mammal and Bird Species Richness in China Based on Habitat Groups
Source: PLoS One. 2015 Dec 2;10(12):e0143996. doi: 10.1371/journal.pone.0143996 (PMC4668080; doi:10.1371/journal.pone.0143996)
Supplement: S5 Table — Six variables that explained most of the variance of species richness were selected based on univariate regression models and hierarchical partitioning. We established the best multivariate model using GLM multivariable regression. To avoid inflation of type I errors and invalid parameter estimate owning to spatial autocorrelation, we then performed SLM multivariate regression (see Methods). Species richness and all continuous variables were log10-transformed (n = 2376; *: Pr(>|z|)<0.05; **: Pr(>|z|)<0.01; ***: Pr(>|z|)<0.001). (DOCX) [file pone.0143996.s009.docx]

**S5 Table. SLM multivariate models for species richness of all mammals and resident birds and treating area as a variable in the models.**

|  | **Variables** |  | **Mammals** | **Resident birds** |
| --- | --- | --- | --- | --- |
| Best model with 6 predictors including area | Mean annual precipitation | z |  | 5.52*** |
|  | Minimum temperature of the coldest month | z |  | 3.01** |
|  | Net primary productivity | z | 7.00*** | 1.76 |
|  | Temperature seasonality | z | -3.67*** | -5.66*** |
|  | Elevation variability | z | 9.72*** | 6.46*** |
|  | Main land cover type | z | -8.61*** |  |
|  | Area | z | 7.55*** | 4.94*** |
|  | AIC |  | 17976 | 21731 |
|  | Fitted values | r^2^ | 0.61 | 0.70 |
|  | Moran’s I |  | -0.01 | -0.03 |
| 20-predictor model | AIC |  | 17985 | 21666 |
|  | Fitted values | r^2^ | 0.61 | 0.71 |

Six variables that explained most of the variance of species richness were selected based on univariate regression models and hierarchical partitioning. We established the best multivariate model using GLM multivariable regression. To avoid inflation of type I errors and invalid parameter estimate owning to spatial autocorrelation, we then performed SLM multivariate regression (see Methods). Species richness and all continuous variables were log10-transformed (n=2376; *: Pr(>|z|)<0.05; **: Pr(>|z|)<0.01; ***: Pr(>|z|)<0.001).
